# Supplementary material for: Heimler Syndrome Is Caused by Hypomorphic Mutations in the Peroxisome-Biogenesis Genes PEX1 and PEX6
Source: Am J Hum Genet. 2015 Sep 17;97(4):535–45. doi: 10.1016/j.ajhg.2015.08.011 (PMC4596894; doi:10.1016/j.ajhg.2015.08.011)
Supplement: Document S1. Figures S1–S4 [file mmc1.pdf]

The American Journal of Human Genetics
Supplemental Data

Heimler Syndrome Is Caused by Hypomorphic Mutations in the Peroxisome-Biogenesis Genes *PEX1* and *PEX6*

Ilham Ratbi, Kim D. Falkenberg, Manou Sommen, Nada Al-Sheqaih, Soukaina Guaoua, Geert Vandeweyer, Jill E. Urquhart, Kate E. Chandler, Simon G. Williams, Neil A. Roberts, Mustapha El Alloussi, Graeme C. Black, Sacha Ferdinandusse, Hind Ramdi, Audrey Heimler, Alan Fryer, Sally-Ann Lynch, Nicola Cooper, Kai Ren Ong, Claire E.L. Smith, Christopher F. Inglehearn, Alan J. Mighell, Claire Elcock, James A. Poulter, Marc Tischkowitz, Sally J. Davies, Abdelaziz Sefiani, Aleksandr A. Mironov, William G. Newman, Hans R. Waterham, and Guy Van Camp

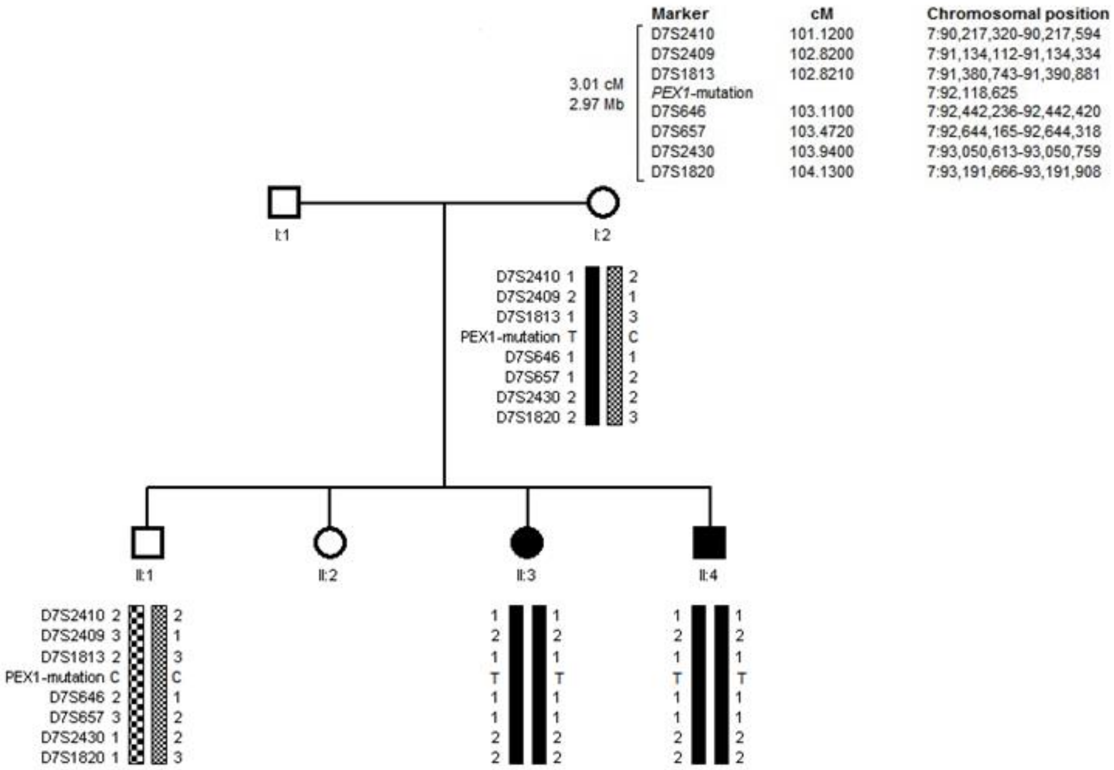

Figure S1. STR genotyping in Family 1. 12 different STR markers in a 3.3 Mb region surrounding *PEX1* were selected and genotyped on all available family members. 5 out of these 12 markers (D7S2313, D7S2796, D7S652, D7S2775, D7S1770) were non-informative. Based on the genotypes of the remaining 7 markers a haplotype was generated, shown below each individual symbol. Black haplotype bars denote the disease-associated haplotype. The genetic map distance in centi-Morgan (cM) and the chromosomal position in base pairs are given behind the marker names.

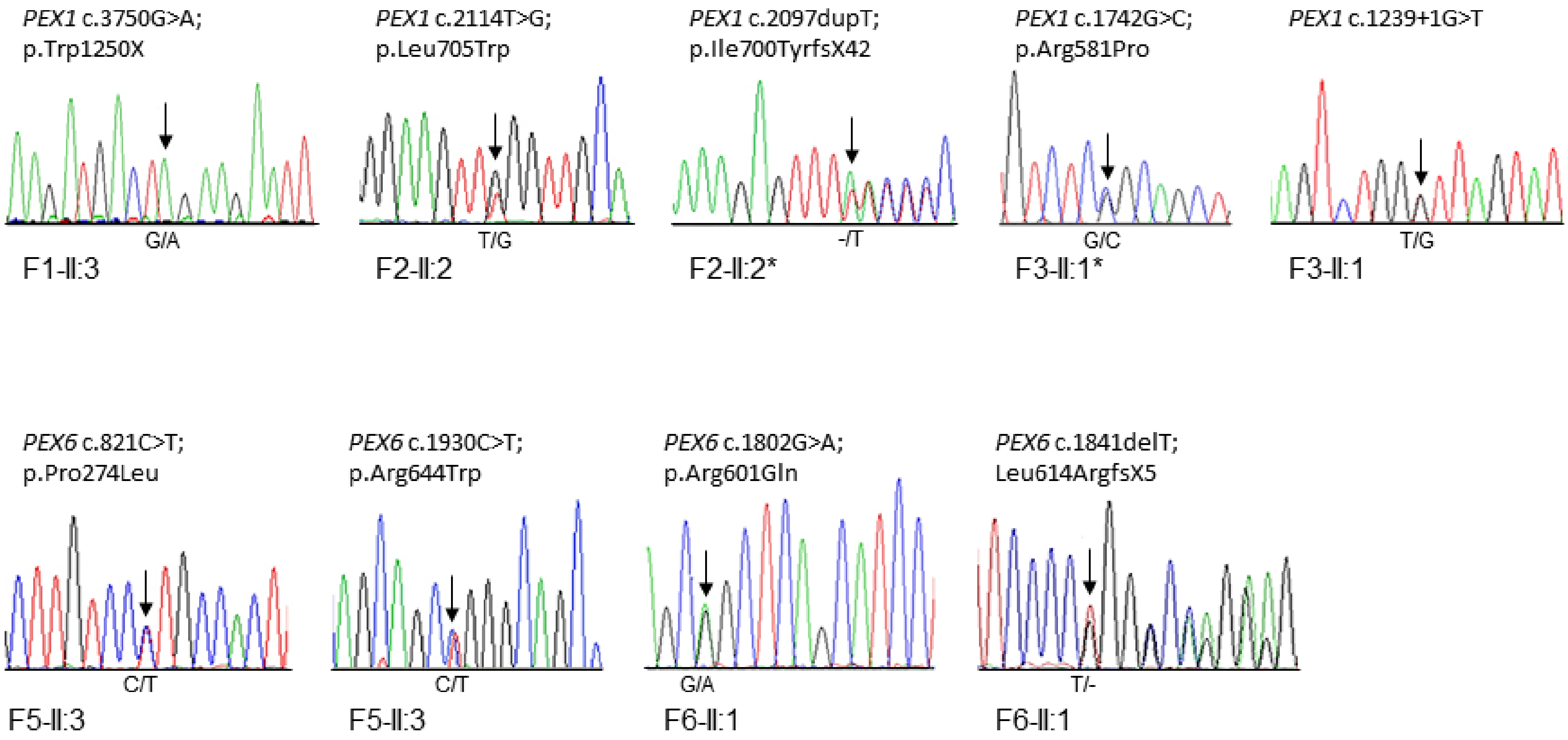

Figure S2. Electropherograms showing mutated sequences of *PEX1* and *PEX6* genes. All mutations had a heterozygous genotype, except for mutation *PEX1* c.3750G>A in individual F1-II:3, which was homozygous. * same variant has been identified in F4.

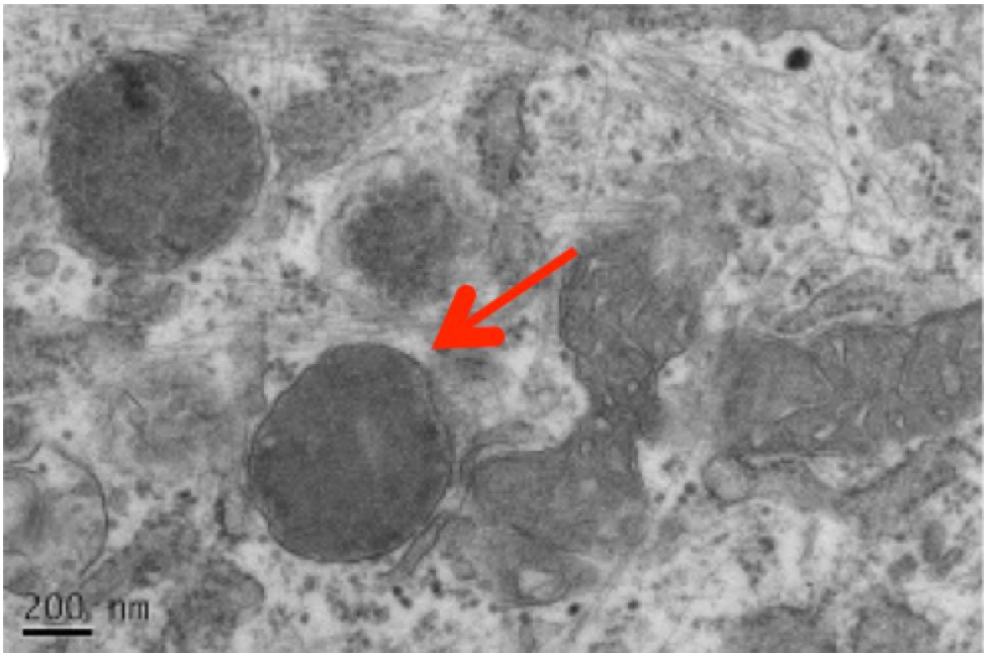

Figure S3. Electron microscopy image of fibroblast cell from individual F5-II:2. Normal morphology of peroxisome indicated by red arrow.

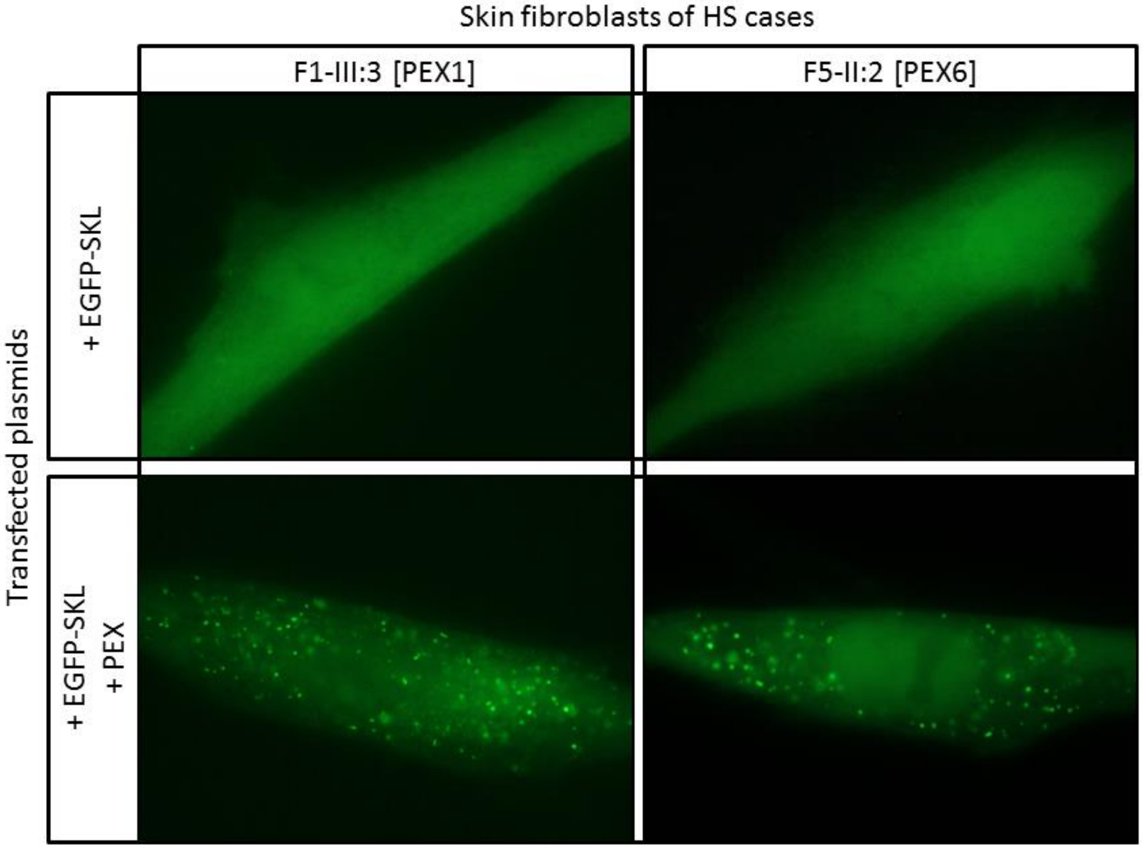

Figure S4. Complementation of peroxisomal defect in HS cells by introduction of PEX1 or PEX6. Skin fibroblasts of HS cases F1-III:3 and F5-II:2 were cultured on 40°C and transfected with a peroxisomal fluorescent marker (EGFP-SKL) and expression vectors containing functional *PEX1* gene copies (for F1-III:3) or *PEX6* gene copies (for F5-II:2). Cells were imaged by fluorescence microscopy and complemented cells (punctate signal) quantified.
