# Supplementary material for: Heimler Syndrome Is Caused by Hypomorphic Mutations in the Peroxisome-Biogenesis Genes PEX1 and PEX6
Source: Am J Hum Genet. 2015 Sep 17;97(4):535–45. doi: 10.1016/j.ajhg.2015.08.011 (PMC4596894; doi:10.1016/j.ajhg.2015.08.011)
Supplement: Document S2. Article plus Supplemental Data [file mmc2.pdf]

Heimler Syndrome Is Caused by Hypomorphic Mutations in the Peroxisome-Biogenesis Genes *PEX1* and *PEX6*

Ilham Ratbi,[1,21] Kim D. Falkenberg,[2,21] Manou Sommen,[3,21] Nada Al-Sheqaih,[4,5] Soukaina Guaoua,[1] Geert Vandeweyer,[3] Jill E. Urquhart,[4,5] Kate E. Chandler,[4,5] Simon G. Williams,[4,5] Neil A. Roberts,[4,5] Mustapha El Alloussi,[6,7] Graeme C. Black,[4,5] Sacha Ferdinandusse,[2] Hind Ramdi,[6] Audrey Heimler,[8] Alan Fryer,[9] Sally-Ann Lynch,[10,11] Nicola Cooper,[12] Kai Ren Ong,[12] Claire E.L. Smith,[13] Christopher F. Inglehearn,[13] Alan J. Mighell,[13,14] Claire Elcock,[15] James A. Poulter,[13] Marc Tischkowitz,[16,17] Sally J. Davies,[18] Abdelaziz Sefiani,[1,19] Aleksandr A. Mironov,[20] William G. Newman,[4,5,22] Hans R. Waterham,[2,22,*] and Guy Van Camp[3,22,*]

Heimler syndrome (HS) is a rare recessive disorder characterized by sensorineural hearing loss (SNHL), amelogenesis imperfecta, nail abnormalities, and occasional or late-onset retinal pigmentation. We ascertained eight families affected by HS and, by using a whole-exome sequencing approach, identified biallelic mutations in *PEX1* or *PEX6* in six of them. Loss-of-function mutations in both genes are known causes of a spectrum of autosomal-recessive peroxisome-biogenesis disorders (PBDs), including Zellweger syndrome. PBDs are characterized by leukodystrophy, hypotonia, SNHL, retinopathy, and skeletal, craniofacial, and liver abnormalities. We demonstrate that each HS-affected family has at least one hypomorphic allele that results in extremely mild peroxisomal dysfunction. Although individuals with HS share some subtle clinical features found in PBDs, the diagnosis was not suggested by routine blood and skin fibroblast analyses used to detect PBDs. In conclusion, our findings define HS as a mild PBD, expanding the pleiotropy of mutations in *PEX1* and *PEX6*.

Introduction

Heimler syndrome (HS [MIM: 234580]) is a rare autosomal-recessive disorder that was first described in 1991 in two siblings, born to healthy and non-consanguineous parents, with sensorineural hearing loss (SNHL), enamel hypoplasia of the secondary dentition, and nail abnormalities.[1] Subsequently, five additional cases have been reported.[2–4] In 2011, Lima et al.[5] reported retinal pigmentation in one of the original individuals diagnosed with HS.[1] The genetic cause of HS had not been identified, but it had been suggested that the syndrome could be due to mutations in a gene affecting derivatives of the ectodermal tissue, given that the described abnormalities have a common embryological origin.[1] In contrast, because of the spectrum of clinical features, Lima et al.[5] classified HS as a ciliopathy. In this study, we ascertained eight HS-affected families and analyzed them in a hypothesis-free way by whole-exome sequencing (WES). We identified biallelic *PEX1* (MIM: 602136) or *PEX6* (MIM: 601498) mutations in six of them. Further functional studies showed that HS is not a ciliopathy but rather a PBD with an atypical mild phenotype that shows limited clinical overlap with other PBDs.

Material and Methods

WES

We used standard techniques[6] to collect blood samples of different family members (Figure 1A) and isolate DNA from blood or saliva. All subjects or their legal representatives provided written informed consent for this study, which was performed in accordance with the Declaration of Helsinki protocols and approved by the local institutional review boards.

WES was undertaken for one or more individuals from families 1–5, 7, and 8. DNA enrichment for WES was achieved with the

[1]Centre de Génomique Humaine, Faculté de Médecine et de Pharmacie, Université Mohammed V, 10100 Rabat, Morocco; [2]Laboratory Genetic Metabolic Diseases, Academic Medical Center, University of Amsterdam, Amsterdam 1105 AZ, the Netherlands; [3]Department of Medical Genetics, University of Antwerp, Antwerp 2610, Belgium; [4]Manchester Centre for Genomic Medicine, St. Mary's Hospital, Manchester Academic Health Sciences Centre, Manchester M13 9WL, UK; [5]Manchester Centre for Genomic Medicine, Institute of Human Development, University of Manchester, Manchester M13 9WL, UK; [6]Département de Pédodontie-Prévention, Faculté de Médecine Dentaire, Université Mohammed V, BP 6212 Madinat Al Irfane, 10100 Rabat, Morocco; [7]Service d'Odontologie, Hôpital Militaire d'Instruction Mohamed V, Avenue des Far, Hay Riad, 10100 Rabat, Morocco; [8]Division of Human Genetics, Schneider Children's Hospital of Long Island Jewish Medical Center, New Hyde Park, NY 11042, USA; [9]Department of Clinical Genetics, Liverpool Women's NHS Foundation Trust, Liverpool L8 7SS, UK; [10]National Centre for Medical Genetics, Our Lady's Children's Hospital, Crumlin, Dublin 12, Ireland; [11]Department of Genetics, Children's University Hospital, Dublin 12, Ireland; [12]West Midlands Regional Genetics Service, Birmingham Women's Hospital NHS Trust, Birmingham B15 2TG, UK; [13]Leeds Institute of Biomedical and Clinical Sciences, St. James's University Hospital, University of Leeds, Leeds LS9 7TF, UK; [14]School of Dentistry, University of Leeds, Leeds LS2 9JT, UK; [15]Academic Unit of Oral Health and Development, School of Clinical Dentistry, University of Sheffield, S10 2TA, UK; [16]Department of Medical Genetics and National Institute for Health Research Cambridge Biomedical Research Centre, University of Cambridge, Cambridge CB2 0QQ, UK; [17]Department of Clinical Genetics, East Anglian Regional Genetics Service, Addenbrooke's Hospital, Cambridge CB2 0QQ, UK; [18]Institute of Medical Genetics, University Hospital of Wales, Cardiff CF14 4XW, UK; [19]Département de Génétique Médicale, Institut National d'Hygiène, BP 769 Agdal, 10090 Rabat, Morocco; [20]Faculty of Life Sciences, University of Manchester, Manchester M13 9PL, UK
[21]These authors contributed equally to this work
[22]These authors contributed equally to this work
*Correspondence: h.r.waterham@amc.uva.nl (H.R.W.), guy.vancamp@uantwerpen.be (G.V.C.)
http://dx.doi.org/10.1016/j.ajhg.2015.08.011. ©2015 The Authors
This is an open access article under the CC BY license (http://creativecommons.org/licenses/by/4.0/).

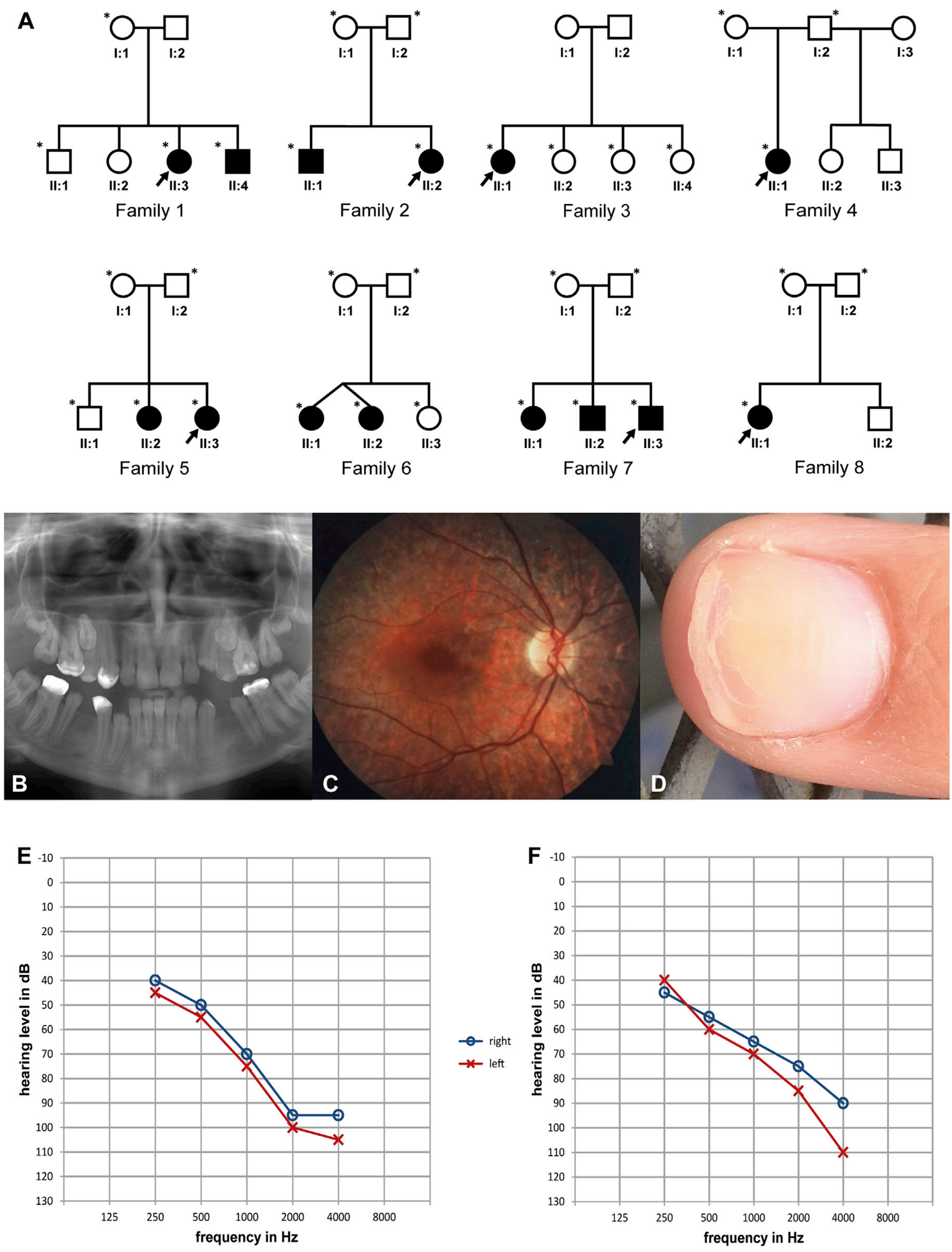

Figure 1. Pedigrees of the Families Affected by HS, Illustration of Clinical Characteristics of Individual F5-II:2, and Air-Conduction Audiograms of HS Individuals

(A) Affected individuals are shaded. Arrows indicate individuals in whom WES was performed and blood and/or fibroblasts were analyzed. For family 1, additional exomes were sequenced for members I:1, II:1, and II:3. DNA for cosegregation analysis was available from family members with an asterisk.

(B) An orthopantogram of individual F5-II:2 shows severe amelogenesis imperfecta.

(C) A retinal photograph taken when individual F5-II:2 was 20 years old shows marked mottling of the retinal pigment epithelium.

(D) A fingernail of individual F5-II:2 shows evidence of onychoschizia and Beau's lines.

(E) An air-conduction audiogram of individual F5-II:2.

(F) An air-conduction audiogram of individual F5-II:3.

Nextera Rapid Capture Exome 38 Mb Kit (Illumina) for family 1 and with the SureSelect Human All Exon Kit v.5 (Agilent) for families 2–5, 7, and 8. Paired-end sequencing (100 bp) was run on an Illumina HiSeq 1500 or 2500. A minimum of 4.5 Gb of sequence was generated for each individual, yielding a mean depth of coverage ranging from 75× to 163× and 88.3%–98% of target bases sequenced at 20× coverage. The sequence data were mapped to the human reference genome (UCSC Genome Browser hg19) with the Burrows Wheeler Aligner (BWA).[7] Variant calling was performed with the Genome Analysis Toolkit (GATK) v.2.4.7.[8] VariantDB was used for variant annotation and filtering. Variant annotation was based on information from GATK, SNPeff,[9] ANNOVAR, and Gene Ontology.[10] Quality-based filtering was performed according to the following parameters: (1) mapping quality above 50, (2) quality by depth above 4.8, (3) mapping-quality rank sum between −3 and 3, and (4) Fisher-scaled strand bias smaller than 20. Next, common variants with a minor allele frequency (MAF) above 1% were filtered out on the basis of dbSNP (v.137), 1000 Genomes (April 2012), and the NHLBI Exome Sequencing Project Exome Variant Server (ESP6500). Variants present in our in-house control database, including 770 exomes, were filtered out. Only non-synonymous, frameshift, nonsense, and splice-site variants and genes containing biallelic variants were selected. The effect of the variant on protein function was predicted by PolyPhen-2, SIFT, and MutationTaster with dbNSFP[11] for non-synonymous variants and with multiple tools for potential splice-site mutations. Confirmation of the putative disease-causing variants and their cosegregation with the disease phenotype and analysis of *PEX6* for variants in family 6[4] were performed by standard Sanger dideoxy sequencing on an ABI 3130XL or ABI 3730 DNA sequencer (Applied Biosystems).

Analysis of STRs and Healthy Control Population for Family 1

For family 1, we amplified, pooled, and analyzed short tandem repeat (STR) markers via capillary electrophoresis on an ABI 3130XL DNA sequencer (Applied Biosystems). We analyzed data with GeneMapper v.3.7 (Applied Biosystems). We used NCBI Map Viewer (annotation release 105) to search for STRs in a 10 Mb region surrounding the *PEX1* mutation.

We collected blood samples from umbilical cords of 250 unrelated newborns originating from different regions of Morocco. The Moroccan origin of their parents and grandparents was confirmed. We obtained informed consent for DNA analysis from the parents. We used a standard salting-out method to extract genomic DNA from 3 ml blood. We developed a real-time PCR (Applied Biosystems 7500 Fast Real-Time PCR Systems) assay by using TaqMan probes for the *PEX1* c.3750G>A (p.Trp1250*) nonsense mutation (Table 2) and validated the assay by using homozygous and heterozygous members of family 1.

Electron Microscopy

We cultured fibroblasts to confluence and fixed them with 4% formaldehyde and 2.5% glutaraldehyde in 0.1 M HEPES buffer (pH 7.2). We postfixed the cells with 1% osmium tetroxide and 1.5% potassium ferrocyanide in 0.1 M cacodylate buffer (pH 7.2) for 1 hr, then in 1% tannic acid in 0.1 M cacodylate buffer (pH 7.2) for 1 hr, and finally in 1% uranyl acetate in water for 1 hr. The samples were dehydrated in ethanol series, infiltrated with TAAB 812 resin, and polymerized for 24 hr at 60°C. Ultrathin sections were cut with a Reichert Ultracut ultramicrotome and visualized with a FEI Tecnai 12 Biotwin microscope at 100 kV accelerating voltage. Images were taken with a Gatan Orius SC1000 CCD camera.

Biochemical and Enzyme-Activity Assays

We measured peroxisomal parameters in plasma (very-long-chain fatty acids [VLCFAs], bile acid intermediates, pipecolic acid, phytanic acid, and pristanic acid),[12] in erythrocytes (plasmalogens),[13] and in skin fibroblasts (VLCFAs,[14] C26:0 and pristanic acid β-oxidation,[15] phytanic acid α-oxidation,[16] and dihydroxyacetonephosphate acyltransferase [DHAPAT] activity[17]). Immunoblot analyses assessed the processing of thiolase and acyl-CoA oxidase I (ACOX1) in fibroblasts.[16,18]

Molecular Cloning

We introduced the different *PEX1* or *PEX6* variants identified in the individuals with HS in the mammalian expression vector pcDNA3 containing full-length *PEX1* or *PEX6* cDNA, respectively, by site-directed mutagenesis (QuikChange Site-Directed Mutagenesis Kit, QIAGEN) according to the manufacturer's instructions. We obtained the pcDNA3 vector containing *PEX6* c.1930C>T (p.Arg644Trp) by amplifying *PEX6* cDNA spanning nucleotides c.1856 to c.*61 (with flanking restriction sites for NheI and KpnI) from total RNA isolated from the fibroblasts of family 5 individual II:2 (F5-II:2). We subsequently subcloned the amplicons into the pcDNA3 vector containing full-length wild-type *PEX6* cDNA. We confirmed successful introduction of the variants by sequence analysis of the cDNAs. To exclude unintentional mutations in the vector backbone during site-directed mutagenesis, we either used several clones per construct for further analyses or recloned the mutated cDNA into pcDNA3 plasmids.

Cell Culture and Transfection

We used primary skin fibroblasts from individuals with HS and primary skin fibroblast cell lines completely deficient of PEX1 (compound heterozygous for p.[Thr263Ilefs*6];[Ile700Tyrfs*42], c.[788_789del];[2097dup])[19] or PEX6 (homozygous for p.Gly135 Aspfs*23 [c.402del]).[20] Cells were cultured in DMEM with L-glutamine (Bio-Whittaker) supplemented with 10% fetal bovine serum (Bio-Whittaker), 25 mM HEPES buffer (BioWhittaker), 100 U/ml penicillin, 100 μg/ml streptomycin (Life Technologies), and 250 ng/ml Fungizone (Life Technologies) in a humidified atmosphere of 5% CO_2 at 37°C or 40°C. Transfections were performed with the AMAXA NHDF Nucleofector Kit (Lonza) according to the manufacturer's instructions (program U23). The medium was changed 24 hr after transfection, and the cells were imaged 72 hr after transfection.

Immunofluorescence Assays

We analyzed peroxisomal appearance in skin fibroblasts from HS individuals by immunofluorescence microscopy. The cells were cultured on glass slides to a confluency of 50%–70%. For fixation, we treated the cells with 2% paraformaldehyde (Merck) in PBS for 20 min at room temperature and permeabilized them with 0.5% Triton X-100 (BioRad) for 5 min. The peroxisomal matrix protein catalase was labeled with the monoclonal antibody α-catalase (Map 17E10, own production), biotinylated α-mouse antibodies (E 433, Dako), and streptavidine-FITC (F 422, Dako). Peroxisomal membranes were labeled with antibodies against PMP70 (ABCD3) (PMP70, no. 718300, Zymogen) and Alexa Fluor 555 goat anti-rabbit (Invitrogen). The slides were fixed on mounting medium

Table 1. Clinical Details of Individuals with HS

	Family 1		Family 2		Family 3	Family 4	Family 5		Family 6		Family 7			Family 8
	II:3	II:4	II:1[1]	II:2[1]	II:1	II:1	II:2	II:3	II:1[4]	II:2[4]	II:1	II:2	II:3	II:1[2]
Origin	Morocco	Morocco	US	US	Ireland	UK	UK	UK	UK	UK	UK	UK	UK	UK
Sex	F	M	M	F	F	F	F	F	F	F	F	M	M	F
Age (years) at last assessment	16	12	31	29	19	24	21	16	21	21	21	20	15	12
Amelogenesis imperfecta	+	+	+	+	+	+	+	+	+	+	+	+	+	+
Intellect	N	N	N	N	N	N	N	N	N	N	N	N	N	N
**SNHL**														
Bilateral or unilateral	B	B	B	B	B	B	B	B	B	B	B	B	B	U
Age (years) of diagnosis	2	1	1.5	2.5	2	1.5	1	0	3	3	6	5	2	7
Degree of hearing loss	S	S	P	P	P	P	P	S	P	P	Mo	Mo	Mo	P
**Nail Abnormalities**														
Beau's lines	–	–	+	+	–	–	+	+	+	+	–	–	–	+
Other nail changes	–	–	L	L	–	–	O	O	L	L	–	–	–	–
**Ocular Features**														
Retinal pigmentation	–	–	+	+	NA	+	+	+	+	+	–	–	–	NA
Macular dystrophy	–	–	+	+	NA	–	–	–	–	–	–	–	–	NA

Abbreviations are as follows: B, bilateral; F, female; L, leukonychia; M, male; Mo, moderate SNHL at high frequencies; N, normal; NA, not assessed; O, onychoschizia; P, profound; S, severe; U, unilateral.

Vectashield H1000 (Brunschwig). Images were taken with the Leica TCS SP8 filter-free spectral confocal microscope.

Assays of Genetic and Functional Complementation

We performed genetic complementation of fibroblasts by transfecting the cells from HS individuals with *PEX* cDNA as described in Ebberink et al.[19] To test the functionality of the PEX variants, we co-transfected pcDNA3-*PEX1* or -*PEX6* plasmids with the peroxisomal matrix marker pEGFP-SKL[21] into skin fibroblasts deficient in PEX1 or PEX6. Cells transfected with only pEGFP-SKL served as negative controls, whereas co-transfections of the marker with pcDNA3 vectors containing the respective wild-type *PEX* cDNA served as positive controls. We subsequently analyzed the localization of the fluorescent signal 3 days after transfection by using the fluorescence microscope Zeiss Axio Observer A1.

To evaluate the effect of the variants found in the affected individuals, we determined per transfection the percentage of cells showing a punctate GFP signal (indicating "peroxisome-positive" or "complemented" cells) of the total number of 100–200 transfected cells. These ratios were normalized to the complementation efficiency of the positive control (set as 100%) and averaged per construct ($n = 5$–7). We used the one-sample Wilcoxon signed-rank test to test the statistical significance of deviations from the positive control.

Results

Identification of *PEX1* and *PEX6* Variants

In order to unravel the genetic cause and the pathological mechanism of HS, we ascertained eight HS-affectd families, including three previously described families (family 2,[1] family 6,[4] and family 8[2]) (Figure 1A and Table 1). The HS individuals were all characterized by a homogeneous phenotype of severe to profound pre-lingual bilateral SNHL most pronounced at high frequencies and amelogenesis imperfecta (Table 1 and Figures 1B–1F). The retinal-pigmentation phenotype was highly variable such that some individuals showed no evidence of retinal pigmentation at 16 and 21 years (in families 1 and 7, respectively), whereas the affected individual in family 4 (F4-II:1) had progressive visual impairment with no peripheral vision and no night vision at age 6 years. Visual assessment was not available from individuals in families 3 and 8. All affected individuals had normal intellect. After the initial WES findings, a single individual (F1-II:3) had a brain MRI scan, which was normal.

We performed WES on at least one affected individual from each family. WES of affected individuals from families 1–5, 7, and 8, as well as selection of biallelic rare or previously reported pathogenic non-synonymous, frameshift, nonsense, or splice-site variants consistent with recessive inheritance co-segregating with the disease phenotype, did not identify variants in a single gene common to all unrelated affected individuals. However, we noted that affected individuals from families 1–4 each had biallelic putative disease-causing variants in *PEX1* (GenBank: NM_000466.2), whereas in family 5 a mutation in *PEX6* (GenBank: NM_000287.3) was found. *PEX1* and *PEX6* are two related genes involved in peroxisome

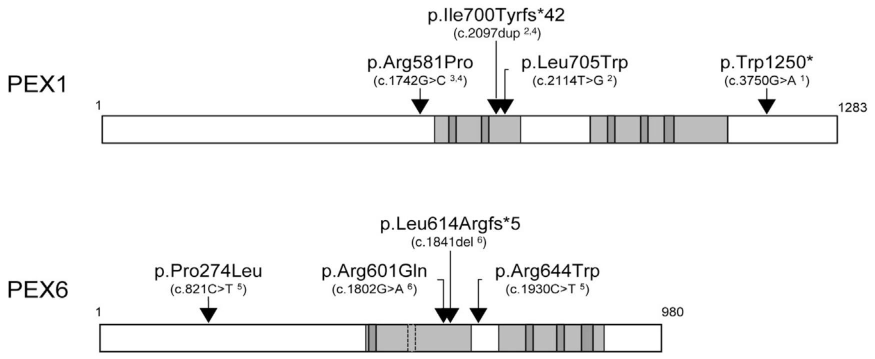

Figure 2. Location of HS-Associated Variants in PEX1 and PEX6
Arrows mark the amino acid positions of the identified alterations. The corresponding position of the alteration at the cDNA level is given between parentheses; the superscript number indicates the family in which the alteration was identified. Gray areas mark the functional AAA domains of the proteins (including the highly conserved Walker motifs in dark gray).

biogenesis (Figure 2). In family 1, we identified a previously unreported homozygous exon 23 nonsense variant, c.3750G>A (p.Trp1250*), only 19 bases from the last exon-exon boundary of *PEX1*. The resultant transcript is assumed to escape nonsense-mediated decay and thus lead to expression of a truncated protein. The homozygous variant was surrounded by a region identical by descent from a common ancestor (Figure S1) and absent in 250 ethnically matched healthy control individuals. In families 2 and 4, we identified a heterozygous, previously reported pathogenic *PEX1* c.2097dup variant resulting in a p.Ile700Tyrfs*42 frameshift[22] in *trans* with a very rare missense variant (in families 2 and 4) on the other allele (Table 2 and Figure S2). In family 3, we identified a pathogenic heterozygous splice-site *PEX1* variant, c.1239+1G>T,[23] also previously reported in individuals with a severe peroxisome-biogenesis disorder (PBD) and in *trans* with an ultra-rare missense variant. In family 5, the two affected individuals were compound heterozygous for a previously reported pathogenic c.821C>T (p.Pro274Leu)[24] variant in *PEX6* and an ultra-rare missense variant on the other allele. We did not perform WES in family 6, but Sanger sequence analysis of *PEX1* and *PEX6* in the affected twins identified a missense variant, c.1802G>A (p.Arg601Gln), in *PEX6* and a single-nucleotide deletion predicted to result in a frameshift on the other allele. The c.1802G>A (p.Arg601Gln) variant has been reported previously[20,23] and is associated with milder PDB phenotypes (data not shown). The variants identified in the individuals with HS and not previously reported were absent in all public databases and in-house databases including 770 exomes. Two missense variants, *PEX1* c.1742G>C and *PEX6* c.1930C>T, were present in the ExAC Browser at a MAF of <0.000033. All variants were confirmed by Sanger sequencing (Figure S2) and predicted to be damaging (Table 2). The variants segregated with the

Table 2. Variants Found in *PEX1* and *PEX6* in Individuals with HS

Family	Gene	Variant	Type of Variant (DNA Level)	Amino Acid Change	SIFT	PolyPhen-2	MutationTaster	Reference
1	*PEX1*[a]	c.3750G>A	nonsense SNV	p.Trp1250*	deleterious	probably damaging	disease causing	not published
2	*PEX1*[a]	c.2114T>G	non-synonymous SNV	p.Leu705Trp	tolerated	probably damaging	disease causing	not published
2 and 4	*PEX1*[a]	c.2097dup	single-nucleotide insertion	p.Ile700Tyrfs*42[b]	NA	NA	disease causing	Collins and Gould[22]
3 and 4	*PEX1*[a]	c.1742G>C	non-synonymous SNV	p.Arg581Pro	deleterious	probably damaging	disease causing	ExAC: 1 in 121,398 alleles
3	*PEX1*[a]	c.1239+1G>T	splice-altering SNV	–	NA	NA	disease causing	Yik et al.[23]
5	*PEX6*[c]	c.821C>T	non-synonymous SNV	p.Pro274Leu	deleterious	benign	disease causing	Steinberg et al.[24]
5	*PEX6*[c]	c.1930C>T	non-synonymous SNV	p.Arg644Trp	deleterious	probably damaging	disease causing	ExAC: 4 in 121,396 alleles
6	*PEX6*[c]	c.1802G>A	non-synonymous SNV	p.Arg601Gln	deleterious	probably damaging	disease causing	Yik et al.[23] Ebberink et al.[19]
6	*PEX6*[c]	c.1841del	single-nucleotide deletion	p.Leu614Argfs*5	NA	NA	disease causing	not published

For each variant, its predicted pathogenetic effect is stated, and in the last column, it is indicated whether the variant has been described before. "Not published" indicates that it has not been published and is also absent from the ExAC database. The following abbreviation is used: NA, not applicable.
[a]Ensembl: ENST00000248633 or GenBank: NM_000466.2.
[b]HUGO nomenclature is based on Ensembl: ENST00000248633 or GenBank: NM_000466.2, which is dissimilar to the protein nomenclature (p.Ile700Tyrfs*41) used in Collins and Gould.[22]
[c]Ensembl: ENST00000304611 or GenBank: NM_000287.3.

Table 3. Peroxisomal Parameters in Blood of Individuals with HS

		Family 1	Family 5	
		II:3	II:2	II:3
**Plasma**				
VLCFA concentration (μmol/l)	C22:0	47.57 (40–119)	NA	NA
	C24:0	38.97 (33–84)	NA	NA
	C26:0	0.67 (0.45–1.32)	3.91 (0.3–4)	3.47 (0.3–4)
VLCFA ratio	C24:0/C22:0	0.82 (0.57–0.92)	0.77 (0.35–1.1)	0.76 (0.35–1.1)
	C26:0/C22:0	0.01 (0–0.02)	0.026 (0.003–0.033)	0.026 (0.003–0.033)
Bile acids (μmol/l)	DHCA	0 (0)	NA	NA
	THCA	0 (0–0.1)	NA	NA
Phytanic acid (μmol/l)		2.8 (0.5–9.9)	5.25 (0–16)	8.1 (0–16)
Pristanic acid (μmol/l)		0.4 (0.1–3)	0.18 (0–5)	1 (0–5)
Pipecolic acid (μmol/l)		1.5 (0.1–7)	NA	NA
**Erythrocytes**				
Plasmalogens (%)	C16:0 DMA	7.7 (6.8–11.9)	4.61 (4.8–12)	5.27 (4.8–12)
	C18:0 DMA	17.5 (10.6–24.9)	9.3 (8.9–27)	10.7 (8.9–27)

Numbers in parentheses indicate the normal range according to the labs in which the assays were performed. Parameters were determined in different labs and reflect different reference values. Abbreviations are as follows: DHCA, 3β,7α-dihydroxycholestanoic acid; DMA, dimethylacetal; NA, not assessed; THCA, 3α,7α,12α-trihydroxycholestanoic acid; VLCFA, very-long-chain fatty acid.

disease phenotype, such that all affected individuals had biallelic variants in *PEX1* or *PEX6*, whereas unaffected siblings and parents were heterozygous or wild-type for the variants. In families 7 and 8, we did not identify a putative candidate gene, and no variants were identified in *PEX1*, *PEX6*, or any of the other currently known *PEX* genes.[25]

Peroxisomal Parameters of Individuals with HS

The finding of biallelic variants in *PEX1* and *PEX6* in the six HS-affected families suggested a peroxisomal defect in the affected individuals. We therefore analyzed plasma and erythrocytes from affected individuals with *PEX1* and *PEX6* variants (F1-II:3, F5-II:2, and F5-II:3) for peroxisomal parameters. These were all within the normal range and did not indicate peroxisomal dysfunction. Moreover, we also did not identify any significant peroxisomal biochemical aberrations in cultured skin fibroblasts from affected individuals F1-II:3 and F5-II:2 (Tables 3 and 4). However, previous studies have shown that individuals with very mild PBDs do not necessarily demonstrate significant biochemical abnormalities in plasma and/or fibroblasts.[26,27] Thus, on the basis of these biochemical findings, we could not exclude a peroxisomal defect. Other evidence of clinical effect due to peroxisome dysfunction was not investigated because there was no clinical indication.

Peroxisomal Studies in the Cultured Fibroblasts of Individuals with HS

Because it is often a more sensitive indicator for a mild PBD,[25,28] we also analyzed the peroxisomal phenotype in the cultured fibroblasts at 37°C from individuals F1-II:3 and F5-II:2 by immunofluorescence (IF) microscopy by using antibodies against the membrane protein PMP70 (ABCD3) and the peroxisomal matrix protein catalase (Figure 3). For both cell lines, we observed a so-called "mosaic" peroxisomal pattern. We saw different types of cells, including cells with normal peroxisomal staining, cells with a reduced number of peroxisomes, and cells with only peroxisomal membrane remnants, referred to as "ghosts," but no import of matrix protein. Peroxisomal mosaicism has been described previously for hypomorphic variants in *PEX1* and *PEX6* and is typically associated with mild PBDs.[29,30] Consistent with cells displaying peroxisomal mosaicism, the cells from individuals F1-II:3 and F5-II:2 showed a more severe peroxisomal phenotype when cultured at an elevated temperature (40°C), and the vast majority of cells lacked catalase-positive peroxisomes (Figure 3). Electron microscopy of fibroblasts cultured at normal temperature revealed no striking ultra-structural abnormalities of peroxisomes (Figure S3).

PEX-cDNA-Transfection Complementation Assay

To confirm that the variants found in *PEX1* and *PEX6* are the cause of the aberrant peroxisomal phenotype, we performed a genetic complementation assay. We cultured fibroblasts of individuals F1-II:3 and F5-II:2 at 40°C and then transfected these with control *PEX1* and *PEX6* cDNAs. The introduction of *PEX1* cDNA rescued the impaired peroxisome biogenesis in cells

Table 4. Peroxisomal Parameters in Primary Skin Fibroblasts of Individuals with HS

		Control Range	Zellweger Spectrum Range	Family 1 II:3	Family 5 II:2
VLCFA concentration (μmol/l)	C22:0	3.84–10.2	2.36–5.59	4.43	3.67
	C24:0	7.76–17.66	5.41–13.39	8.36	8.58
	C26:0	0.18–0.38	0.59–3.38	0.24	0.22
VLCFA ratio	C24:0/C22:0	1.55–2.3	2.08–3.4	1.89	2.34
	C26:0/C22:0	0.03–0.07	0.11–1.17	0.05	0.06
DHAPAT activity [nmol/(2h × mg protein)]		5.4–10.6	0.1–0.9	7.1	14
ACOX1 immunoblot	72 kDa	+	+	+	+
	52 kDa	+	−	+	+
	20 kDa	++	−	+	+
Thiolase immunoblot	44 kDa	−	+	−	−
	41 kDa	+	−	+	+
α oxidation activity [pmol/(h × mg protein)]	phytanic acid substrate	28–95	0–10	34	39
β oxidation activity [pmol/(h × mg protein)]	C16:0 substrate	3,330–7,790	3,330–7,790	7,162	5,959
	C26:0 substrate	800–2,040	50–350	1,088	1,013
	pristanic acid substrate	790–1,690	0–30	1,092	756

Abbreviations are as follows: ACOX1, acyl-CoA oxidase I; DHAPAT, dihydroxyacetonephosphate acyltransferase; VLCFA, very-long-chain fatty acid.

from individual F1-II:3, and *PEX6* cDNA rescued peroxisome biogenesis in cells from individual F5-II:2 (complementation in 14% or 20% of cells, respectively; Figure S4). These results confirm that the variants in *PEX1* and *PEX6* are the cause of the peroxisomal defects in the individuals with HS.

To determine whether, and to which degree, each of the identified *PEX1* and *PEX6* variants affect peroxisome biogenesis, we co-transfected cDNAs harboring the different variants with fluorescent peroxisomal marker EGFP-SKL into cells completely deficient in PEX1 or PEX6. We compared the capability of these cDNAs to functionally complement the peroxisome-deficient cells to the complementation capability of the control cDNAs (Figure 4). Because *PEX1* c.1239+1G>T causes a splicing defect and consequently does not produce a functional protein, we did not test it in the complementation assay. Transfection with the constructs containing the previously reported pathogenic variants *PEX1* c.2097dup and *PEX6* c.821C>T, as well as the variant *PEX6* c.1841del, resulted in no (0%) to minimal (1.3%) functional complementation. These results are consistent with the severe clinical presentation and complete absence of import of peroxisomal matrix protein in individuals homozygous for these variants[31] (data not shown). In contrast, transfection with the other variants resulted in rescue of peroxisomal biogenesis in between 23% and 58% of cells, indicating that these variants are associated with significant residual activity. Thus, all affected individuals possess at least one PEX variant with residual activity in peroxisomal biogenesis.

Discussion

PEX1 and *PEX6* encode two interacting proteins that belong to the peroxisomal import machinery and that are involved in the shuttling of PEX5, the cytosolic receptor for peroxisomal matrix proteins.[32,33] Biallelic pathogenic variants in *PEX1*, *PEX5*, *PEX6*, or any of the other 11 *PEX* genes result in a PBD, which is characterized by defective peroxisome assembly due to impaired import of proteins into the peroxisomal matrix or membrane.[25] Peroxisomes are found in virtually all human cells and play a crucial role in a number of metabolic pathways.[34] PBDs usually have a severe progressive multi-systemic clinical presentation, including developmental delay, seizures, SNHL, retinopathy, peripheral neuropathy, leukodystrophy, and skeletal, craniofacial, and liver abnormalities.[35–37] Dependent on the underlying genetic defect, however, the clinical presentation and survival of individuals with a PBD can show a wide variability ranging from the severe, early-childhood lethal Zellweger syndrome to milder phenotypes, including isolated progressive ataxia.[26,27] To reflect this clinical variability, the PBDs are often referred to as Zellweger spectrum disorders. Accounting for 60% and 16%, respectively, of diagnosed cases,[19] mutations in *PEX1* and *PEX6* represent the most common causes of PBDs. Our combined findings show that HS is caused by compound heterozygosity for a loss-of-function allele and a hypomorphic allele in *PEX1* or *PEX6*. Alternatively, in family 1 a homozygous hypomorphic allele also results in the HS phenotype. Consequently, although PBD-affected individuals with mild or normal

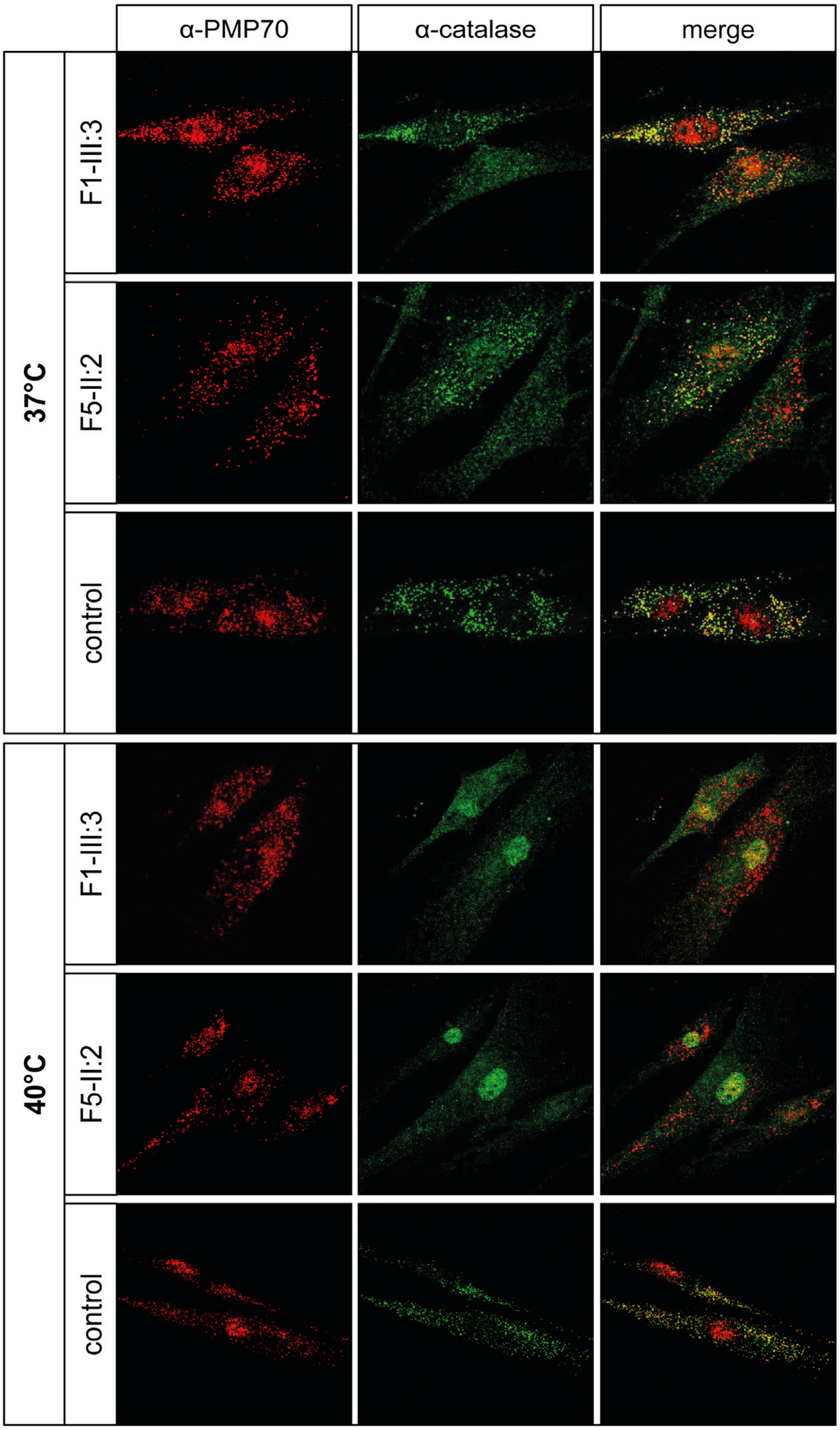

Figure 3. Peroxisomal Phenotype of Cells from Individuals with HS

Skin fibroblasts of individuals F1-III:3 and F5-II:2 and control fibroblasts were cultured at 37°C or 40°C and immunolabeled for PMP70 (ABCD3) (red, peroxisomal membrane protein) and catalase (green, peroxisomal matrix protein). In cells of individuals with HS, the staining at 37°C revealed a mosaic pattern showing cells with a normal peroxisomal phenotype (positive for both PMP70 and catalase) and cells with import-incompetent peroxisomes ("ghosts;" positive for PMP70 and negative for catalase). At 40°C, the vast majority of these cells showed import-incompetent peroxisomes (positive for PMP70 and negative for catalase). Control cells showed a normal peroxisomal phenotype in all conditions.

peroxisome functions in blood and fibroblasts and normal intellect have been described before, HS represents a discrete phenotypic entity at the mildest end of the PBD clinical spectrum. The characteristic presentation overlaps some of the clinical features observed in affected individuals with PBDs.[38,39] Indeed, SNHL is a common feature of PBDs, and tooth and nail abnormalities have been described in PBD-affected individuals with prolonged survival, but always in association with additional and more severe features.[40–44] Importantly, in contrast to individuals with PBDs at the severe end of the clinical spectrum, the individuals with HS showed no identifiable dysmorphic or additional neurological features. Other evidence of clinical effect due to peroxisome dysfunction was not investigated, given that there was no clinical indication. However, future assessments of individuals with HS should consider other features well described in individuals with PBDs, including brain imaging, testing for adrenal insufficiency, liver-function tests, and clinical assessment for evidence of (progressive) peripheral neuropathy. Compound heterozygosity of the hypomorphic *PEX6* c.1802G>A allele has been reported previously in seven individuals with a Zellweger spectrum disorder.[20,23] In all reported individuals, the p.Arg601Gln allele was in *trans* with a severe *PEX6* allele that, when homozygous or in *trans* with another severe *PEX6* allele, causes a severe peroxisomal phenotype (six individuals) or that is predicted to be deleterious (one individual). For three of the seven individuals, who were diagnosed at an advanced adult age, studies in fibroblasts revealed peroxisomal mosaicism at 37°C and slightly elevated C26:C22 levels (unpublished results). Other peroxisomal parameters were normal. No clinical data are available for determining whether these individuals showed a HS-like phenotype. However, these findings suggest that the *PEX6* c.1802G>A allele is a risk allele for mild PBD when in *trans* with a severe *PEX6* allele. Because

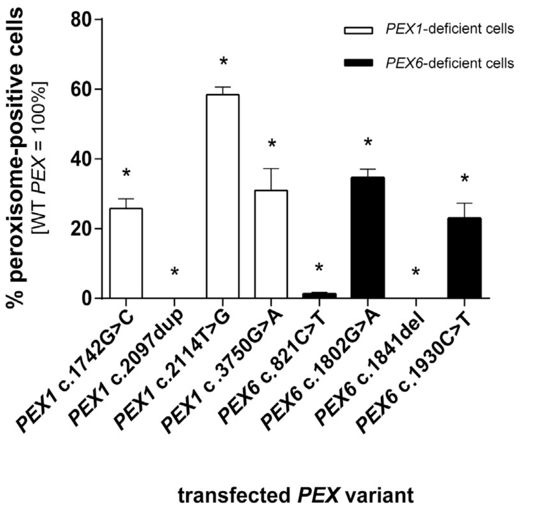

Figure 4. Effect of the *PEX1* and *PEX6* Mutations on Peroxisome Biogenesis

Fibroblasts deficient in *PEX1* or *PEX6* were transfected with a peroxisomal fluorescent marker and expression plasmids containing the constructed *PEX* variants. The ratio of complemented cells was quantified per construct and normalized to the maximal complementation capability as measured in co-transfections of peroxisomal markers with the functional *PEX* versions (positive controls set as 100%). n = 5–7 per construct (mean ± SEM); *$p < 0.05$ (statistical analysis by one-sample Wilcoxon signed-rank test for which the null hypothesis, H_0, was that the median sample value equals the maximal complementation capability [100%], which is the ratio of peroxisome-positive cells to wild-type *PEX*-complemented cells).

the *PEX6* c.1802G>A allele has a frequency of 0.41% in the European population (see ExAC Browser in the Web Resources), we expect that future WES studies will identify additional individuals who have a mild PBD due to compound heterozygosity of the *PEX6* c.1802G>A allele and a severe *PEX6* allele and who have not been suspected of or analyzed for a peroxisomal disorder on the basis of clinical diagnosis.

Because standard biochemical screening of blood for evidence of a peroxisomal disorder would not have provided a diagnosis in the individuals with HS, our study used a genomic approach to diagnose a rare inborn error of metabolism. Our findings could also be relevant for the development of future therapy for PBDs, because they indicate that partial restoration of the function of altered PEX proteins would lead to a phenotype consistent with HS. Notably, the normal intellectual development and lack of severe hepatic and neurological impairment should be instructive in the expected outcomes of future therapeutic trials. Because of the SNHL and retinal pigmentation, HS is also an important differential diagnosis for Usher syndrome (MIM: 276900). Our study allows precise molecular differentiation of the two diagnoses and indicates that all individuals with SNHL and retinal pigmentation are candidates for mutation analysis of *PEX* genes. Our data indicate that HS is a clinically and genetically heterogeneous condition due to biallelic variants in *PEX1* or *PEX6*. We did not identify *PEX* variants in families 7 or 8, nor did we find another genetic cause. However, there are phenotypic differences between these families and those in whom *PEX1* or *PEX6* variants were identified. Although amelogenesis imperfecta was present in all affected individuals in families 7 and 8, the SNHL in the individuals in family 7 was less severe than in the other affected individuals. In the previously reported affected individual in family 8, the SNHL was unilateral, whereas it was bilateral in all the other affected individuals. In addition, only individual F8-II:1 presented with subtle Beau's lines, individuals in family 7 did not show any nail abnormalities, and ocular features were not present in any of the individuals in families 7 or 8. So, these phenotypic differences might account for the lack of *PEX1* or *PEX6* mutations, indicating both clinical and genetic heterogeneity.

The development of genomic medicine has stimulated an active debate about the interpretation of sequence variants and the challenges of pleiotropy.[45] Our data highlight the complexity of the clinical interpretation of genomic data by showing that different mutations in *PEX1* and *PEX6* result in strikingly different clinical outcomes. In addition, the results of this study further emphasize the power of functional laboratory tests in the evaluation of rare variants in known disease-associated genes.

Supplemental Data

Supplemental Data include four figures and can be found with this article online at http://dx.doi.org/10.1016/j.ajhg.2015.08.011.

Acknowledgments

We thank the families for their participation in this study. We acknowledge Dr. Nawfal Fejjal for his participation in the clinical investigations of the Moroccan family. This study was supported by funding from the Belgian Science Policy Office Interuniversity Attraction Poles (BELSPO-IAP) program (project IAP P7/43-BeMGI to G.V.C.) and funding from the Marie Curie Initial Training Networks action (FP7-2012-PERFUME-316723 to K.D.F. and H.R.W.). We acknowledge support from the Manchester Biomedical Research Centre. This study was supported by funding from the Wellcome Trust (grant no. 093113 to C.F.I. and A.J.M.). C.F.I., G.C.B., and J.A.P. are members of the UK Inherited Retinal Disease Consortium, supported by RP Fighting Blindness and Fight For Sight (RP Genome Project GR586). The authors would like to thank the Exome Aggregation Consortium and the groups that provided exome variant data for comparison. A full list of contributing groups can be found at http://exac.broadinstitute.org/about.

Received: April 24, 2015
Accepted: August 21, 2015
Published: September 17, 2015

Web Resources

The URLs for data presented herein are as follows:

1000 Genomes, http://www.1000genomes.org/
ANNOVAR, http://www.openbioinformatics.org/annovar/

dbSNP, http://www.ncbi.nlm.nih.gov/SNP/
Exome Aggregation Consortium (ExAC) Browser, http://exac.broadinstitute.org
NCBI Map Viewer, http://www.ncbi.nlm.nih.gov/mapview/
NHLBI Exome Sequencing Project (ESP) Exome Variant Server, http://evs.gs.washington.edu/EVS/
OMIM, http://www.omim.org/
UCSC Genome Browser, https://genome.ucsc.edu/index.html
VariantDB, http://www.biomina.be/app/variantdb

References

1. Heimler, A., Fox, J.E., Hershey, J.E., and Crespi, P. (1991). Sensorineural hearing loss, enamel hypoplasia, and nail abnormalities in sibs. Am. J. Med. Genet. *39*, 192–195.
2. Tischkowitz, M., Clenaghan, C., Davies, S., Hunter, L., Potts, J., and Verhoef, S. (1999). Amelogenesis imperfecta, sensorineural hearing loss, and Beau's lines, a second case report of Heimler's syndrome. J. Med. Genet. *36*, 941–943.
3. Pollak, C., Floy, M., and Say, B. (2003). Sensorineural hearing loss and enamel hypoplasia with subtle nail findings: another family with Heimler's syndrome. Clin. Dysmorphol. *12*, 55–58.
4. Ong, K.R., Visram, S., McKaig, S., and Brueton, L.A. (2006). Sensorineural deafness, enamel abnormalities and nail abnormalities: a case report of Heimler syndrome in identical twin girls. Eur. J. Med. Genet. *49*, 187–193.
5. Lima, L.H., Barbazetto, I.A., Chen, R., Yannuzzi, L.A., Tsang, S.H., and Spaide, R.F. (2011). Macular dystrophy in Heimler syndrome. Ophthalmic Genet. *32*, 97–100.
6. Miller, S.A., Dykes, D.D., and Polesky, H.F. (1988). A simple salting out procedure for extracting DNA from human nucleated cells. Nucleic Acids Res. *16*, 1215.
7. Li, H., and Durbin, R. (2009). Fast and accurate short read alignment with Burrows-Wheeler transform. Bioinformatics *25*, 1754–1760.
8. McKenna, A., Hanna, M., Banks, E., Sivachenko, A., Cibulskis, K., Kernytsky, A., Garimella, K., Altshuler, D., Gabriel, S., Daly, M., and DePristo, M.A. (2010). The Genome Analysis Toolkit: a MapReduce framework for analyzing next-generation DNA sequencing data. Genome Res. *20*, 1297–1303.
9. Cingolani, P., Platts, A., Wang, L., Coon, M., Nguyen, T., Wang, L., Land, S.J., Lu, X., and Ruden, D.M. (2012). A program for annotating and predicting the effects of single nucleotide polymorphisms, SnpEff: SNPs in the genome of Drosophila melanogaster strain w1118; iso-2; iso-3. Fly (Austin) *6*, 80–92.
10. Ashburner, M., Ball, C.A., Blake, J.A., Botstein, D., Butler, H., Cherry, J.M., Davis, A.P., Dolinski, K., Dwight, S.S., Eppig, J.T., et al.; The Gene Ontology Consortium (2000). Gene ontology: tool for the unification of biology. Nat. Genet. *25*, 25–29.
11. Liu, X., Jian, X., and Boerwinkle, E. (2011). dbNSFP: a lightweight database of human nonsynonymous SNPs and their functional predictions. Hum. Mutat. *32*, 894–899.
12. Dacremont, G., Cocquyt, G., and Vincent, G. (1995). Measurement of very long-chain fatty acids, phytanic and pristanic acid in plasma and cultured fibroblasts by gas chromatography. J. Inherit. Metab. Dis. *18* (*Suppl 1*), 76–83.
13. Dacremont, G., and Vincent, G. (1995). Assay of plasmalogens and polyunsaturated fatty acids (PUFA) in erythrocytes and fibroblasts. J. Inherit. Metab. Dis. *18* (*Suppl 1*), 84–89.
14. Vreken, P., van Lint, A.E., Bootsma, A.H., Overmars, H., Wanders, R.J., and van Gennip, A.H. (1998). Rapid stable isotope dilution analysis of very-long-chain fatty acids, pristanic acid and phytanic acid using gas chromatography-electron impact mass spectrometry. J. Chromatogr. B Biomed. Sci. Appl. *713*, 281–287.
15. Wanders, R.J., Denis, S., Ruiter, J.P., Schutgens, R.B., van Roermund, C.W., and Jacobs, B.S. (1995). Measurement of peroxisomal fatty acid beta-oxidation in cultured human skin fibroblasts. J. Inherit. Metab. Dis. *18* (*Suppl 1*), 113–124.
16. Wanders, R.J., and Van Roermund, C.W. (1993). Studies on phytanic acid alpha-oxidation in rat liver and cultured human skin fibroblasts. Biochim. Biophys. Acta *1167*, 345–350.
17. Ofman, R., and Wanders, R.J. (1994). Purification of peroxisomal acyl-CoA: dihydroxyacetonephosphate acyltransferase from human placenta. Biochim. Biophys. Acta *1206*, 27–34.
18. van Grunsven, E.G., van Berkel, E., Mooijer, P.A., Watkins, P.A., Moser, H.W., Suzuki, Y., Jiang, L.L., Hashimoto, T., Hoefler, G., Adamski, J., and Wanders, R.J. (1999). Peroxisomal bifunctional protein deficiency revisited: resolution of its true enzymatic and molecular basis. Am. J. Hum. Genet. *64*, 99–107.
19. Ebberink, M.S., Mooijer, P.A., Gootjes, J., Koster, J., Wanders, R.J., and Waterham, H.R. (2011). Genetic classification and mutational spectrum of more than 600 patients with a Zellweger syndrome spectrum disorder. Hum. Mutat. *32*, 59–69.
20. Ebberink, M.S., Kofster, J., Wanders, R.J., and Waterham, H.R. (2010). Spectrum of PEX6 mutations in Zellweger syndrome spectrum patients. Hum. Mutat. *31*, E1058–E1070.
21. Waterham, H.R., Koster, J., van Roermund, C.W., Mooyer, P.A., Wanders, R.J., and Leonard, J.V. (2007). A lethal defect of mitochondrial and peroxisomal fission. N. Engl. J. Med. *356*, 1736–1741.
22. Collins, C.S., and Gould, S.J. (1999). Identification of a common PEX1 mutation in Zellweger syndrome. Hum. Mutat. *14*, 45–53.
23. Yik, W.Y., Steinberg, S.J., Moser, A.B., Moser, H.W., and Hacia, J.G. (2009). Identification of novel mutations and sequence variation in the Zellweger syndrome spectrum of peroxisome biogenesis disorders. Hum. Mutat. *30*, E467–E480.
24. Steinberg, S., Chen, L., Wei, L., Moser, A., Moser, H., Cutting, G., and Braverman, N. (2004). The PEX Gene Screen: molecular diagnosis of peroxisome biogenesis disorders in the Zellweger syndrome spectrum. Mol. Genet. Metab. *83*, 252–263.
25. Waterham, H.R., and Ebberink, M.S. (2012). Genetics and molecular basis of human peroxisome biogenesis disorders. Biochim. Biophys. Acta *1822*, 1430–1441.
26. Zeharia, A., Ebberink, M.S., Wanders, R.J., Waterham, H.R., Gutman, A., Nissenkorn, A., and Korman, S.H. (2007). A novel PEX12 mutation identified as the cause of a peroxisomal biogenesis disorder with mild clinical phenotype, mild biochemical abnormalities in fibroblasts and a mosaic catalase immunofluorescence pattern, even at 40 degrees C. J. Hum. Genet. *52*, 599–606.
27. Régal, L., Ebberink, M.S., Goemans, N., Wanders, R.J., De Meirleir, L., Jaeken, J., Schrooten, M., Van Coster, R., and Waterham, H.R. (2010). Mutations in PEX10 are a cause of autosomal recessive ataxia. Ann. Neurol. *68*, 259–263.
28. Ebberink, M.S., Koster, J., Visser, G., Spronsen, Fv., Stolte-Dijkstra, I., Smit, G.P., Fock, J.M., Kemp, S., Wanders, R.J.,

and Waterham, H.R. (2012). A novel defect of peroxisome division due to a homozygous non-sense mutation in the PEX11β gene. J. Med. Genet. *49*, 307–313.
29. Gootjes, J., Schmohl, F., Mooijer, P.A., Dekker, C., Mandel, H., Topcu, M., Huemer, M., Von Schütz, M., Marquardt, T., Smeitink, J.A., et al. (2004). Identification of the molecular defect in patients with peroxisomal mosaicism using a novel method involving culturing of cells at 40 degrees C: implications for other inborn errors of metabolism. Hum. Mutat. *24*, 130–139.
30. Imamura, A., Shimozawa, N., Suzuki, Y., Zhang, Z., Tsukamoto, T., Fujiki, Y., Orii, T., Osumi, T., and Kondo, N. (2000). Restoration of biochemical function of the peroxisome in the temperature-sensitive mild forms of peroxisome biogenesis disorder in humans. Brain Dev. *22*, 8–12.
31. Maxwell, M.A., Allen, T., Solly, P.B., Svingen, T., Paton, B.C., and Crane, D.I. (2002). Novel PEX1 mutations and genotype-phenotype correlations in Australasian peroxisome biogenesis disorder patients. Hum. Mutat. *20*, 342–351.
32. Fujiki, Y., Nashiro, C., Miyata, N., Tamura, S., and Okumoto, K. (2012). New insights into dynamic and functional assembly of the AAA peroxins, Pex1p and Pex6p, and their membrane receptor Pex26p in shuttling of PTS1-receptor Pex5p during peroxisome biogenesis. Biochim. Biophys. Acta *1823*, 145–149.
33. Tamura, S., Matsumoto, N., Takeba, R., and Fujiki, Y. (2014). AAA peroxins and their recruiter Pex26p modulate the interactions of peroxins involved in peroxisomal protein import. J. Biol. Chem. *289*, 24336–24346.
34. Wanders, R.J., and Waterham, H.R. (2006). Biochemistry of mammalian peroxisomes revisited. Annu. Rev. Biochem. *75*, 295–332.
35. Braverman, N.E., D'Agostino, M.D., and Maclean, G.E. (2013). Peroxisome biogenesis disorders: Biological, clinical and pathophysiological perspectives. Dev. Disabil. Res. Rev. *17*, 187–196.
36. Aubourg, P., and Wanders, R. (2013). Peroxisomal disorders. Handb. Clin. Neurol. *113*, 1593–1609.
37. Poll-The, B.T., and Gärtner, J. (2012). Clinical diagnosis, biochemical findings and MRI spectrum of peroxisomal disorders. Biochim. Biophys. Acta *1822*, 1421–1429.
38. Majewski, J., Wang, Z., Lopez, I., Al Humaid, S., Ren, H., Racine, J., Bazinet, A., Mitchel, G., Braverman, N., and Koenekoop, R.K. (2011). A new ocular phenotype associated with an unexpected but known systemic disorder and mutation: novel use of genomic diagnostics and exome sequencing. J. Med. Genet. *48*, 593–596.
39. Raas-Rothschild, A., Wanders, R.J., Mooijer, P.A., Gootjes, J., Waterham, H.R., Gutman, A., Suzuki, Y., Shimozawa, N., Kondo, N., Eshel, G., et al. (2002). A PEX6-defective peroxisomal biogenesis disorder with severe phenotype in an infant, versus mild phenotype resembling Usher syndrome in the affected parents. Am. J. Hum. Genet. *70*, 1062–1068.
40. Poll-The, B.T., Gootjes, J., Duran, M., De Klerk, J.B., Wenniger-Prick, L.J., Admiraal, R.J., Waterham, H.R., Wanders, R.J., and Barth, P.G. (2004). Peroxisome biogenesis disorders with prolonged survival: phenotypic expression in a cohort of 31 patients. Am. J. Med. Genet. A. *126A*, 333–338.
41. Dursun, A., Gucer, S., Ebberink, M.S., Yigit, S., Wanders, R.J., and Waterham, H.R. (2009). Zellweger syndrome with unusual findings: non-immune hydrops fetalis, dermal erythropoiesis and hypoplastic toe nails. J. Inherit. Metab. Dis. *32* (*Suppl 1*), S345–S348.
42. Acharya, B.S., Ritwik, P., Velasquez, G.M., and Fenton, S.J. (2012). Medical-dental findings and management of a child with infantile Refsum disease: a case report. Spec. Care Dentist. *32*, 112–117.
43. Tran, D., Greenhill, W., and Wilson, S. (2011). Infantile refsum disease with enamel defects: a case report. Pediatr. Dent. *33*, 266–270.
44. Bader, P.I., Dougherty, S., Cangany, N., Raymond, G., and Jackson, C.E. (2000). Infantile refsum disease in four Amish sibs. Am. J. Med. Genet. *90*, 110–114.
45. Kocarnik, J.M., and Fullerton, S.M. (2014). Returning pleiotropic results from genetic testing to patients and research participants. JAMA *311*, 795–796.

The American Journal of Human Genetics
Supplemental Data

Heimler Syndrome Is Caused by Hypomorphic Mutations in the Peroxisome-Biogenesis Genes *PEX1* and *PEX6*

Ilham Ratbi, Kim D. Falkenberg, Manou Sommen, Nada Al-Sheqaih, Soukaina Guaoua, Geert Vandeweyer, Jill E. Urquhart, Kate E. Chandler, Simon G. Williams, Neil A. Roberts, Mustapha El Alloussi, Graeme C. Black, Sacha Ferdinandusse, Hind Ramdi, Audrey Heimler, Alan Fryer, Sally-Ann Lynch, Nicola Cooper, Kai Ren Ong, Claire E.L. Smith, Christopher F. Inglehearn, Alan J. Mighell, Claire Elcock, James A. Poulter, Marc Tischkowitz, Sally J. Davies, Abdelaziz Sefiani, Aleksandr A. Mironov, William G. Newman, Hans R. Waterham, and Guy Van Camp

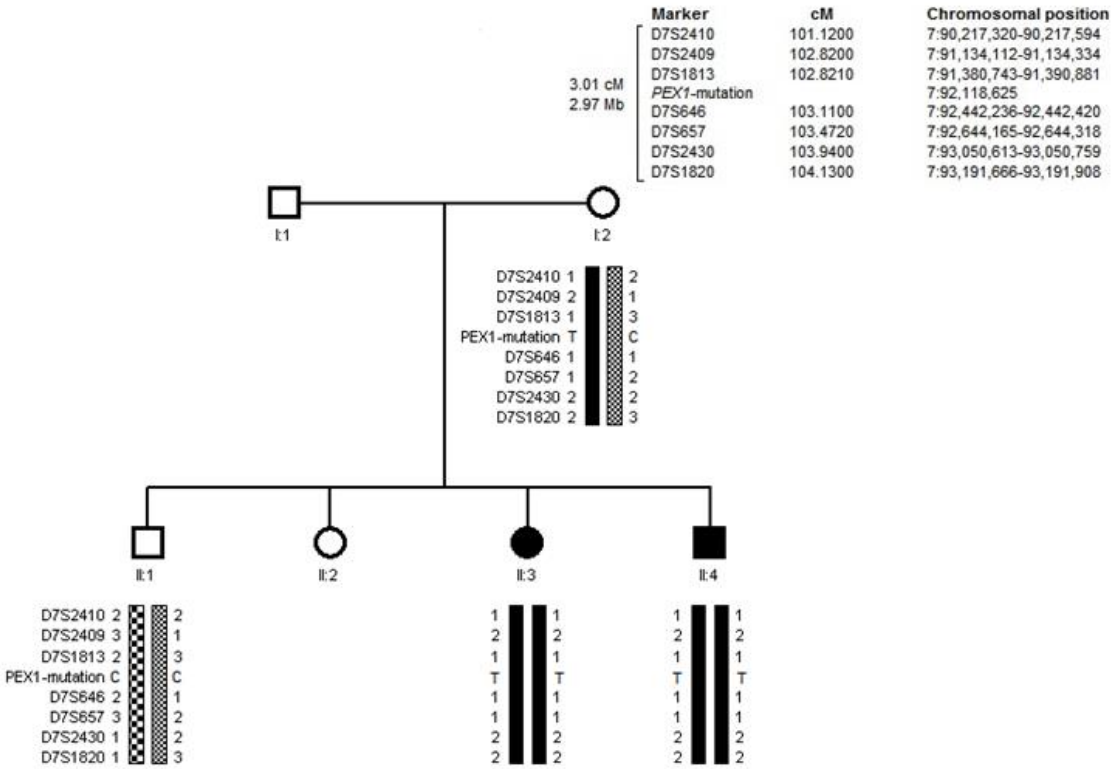

Figure S1. STR genotyping in Family 1. 12 different STR markers in a 3.3 Mb region surrounding *PEX1* were selected and genotyped on all available family members. 5 out of these 12 markers (D7S2313, D7S2796, D7S652, D7S2775, D7S1770) were non-informative. Based on the genotypes of the remaining 7 markers a haplotype was generated, shown below each individual symbol. Black haplotype bars denote the disease-associated haplotype. The genetic map distance in centi-Morgan (cM) and the chromosomal position in base pairs are given behind the marker names.

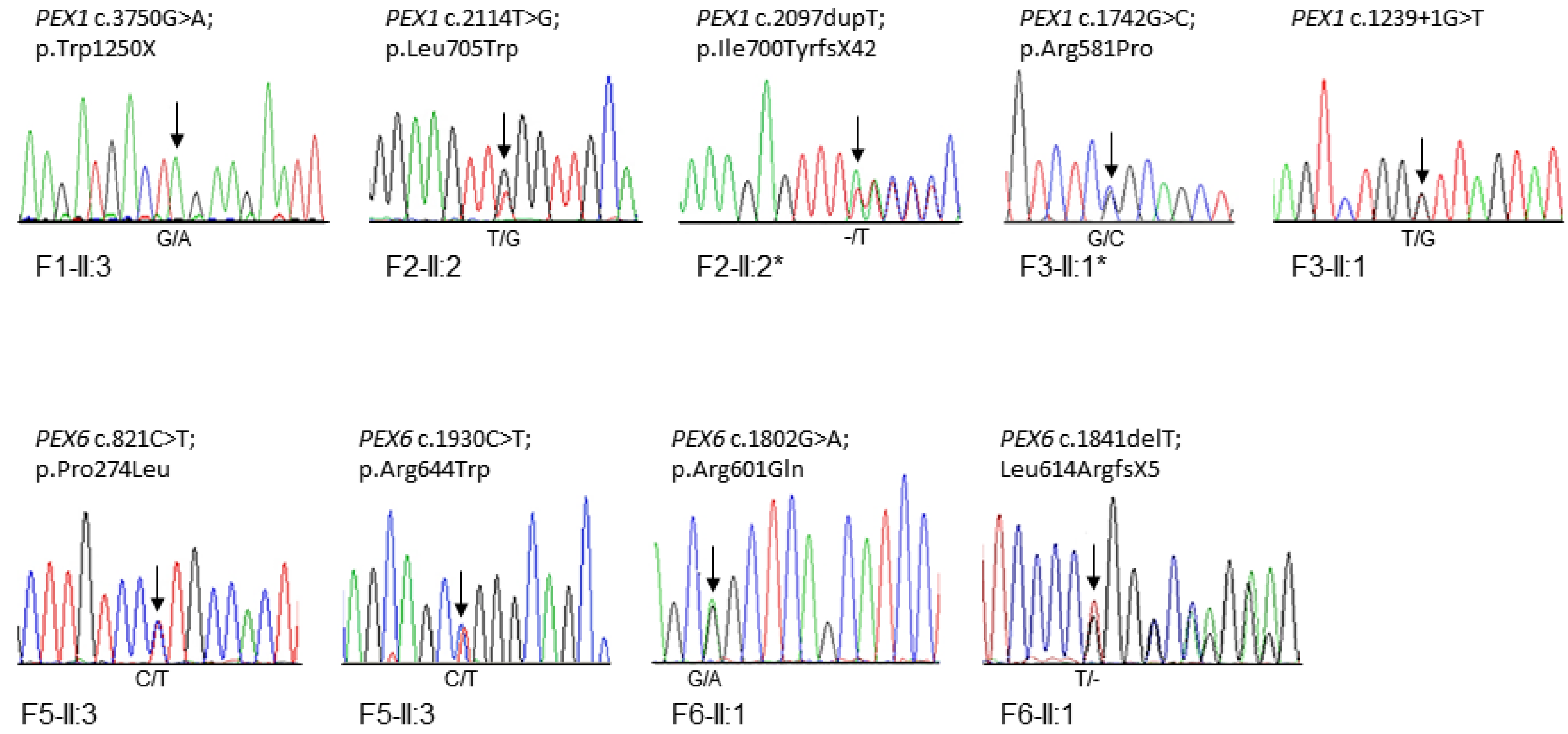

Figure S2. Electropherograms showing mutated sequences of *PEX1* and *PEX6* genes. All mutations had a heterozygous genotype, except for mutation *PEX1* c.3750G>A in individual F1-II:3, which was homozygous. * same variant has been identified in F4.

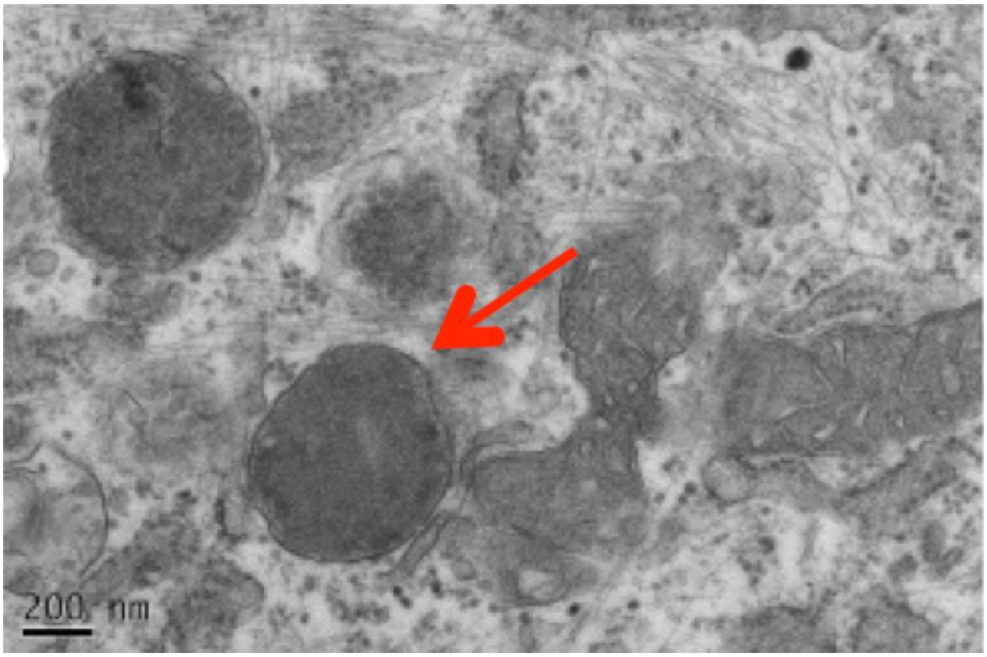

Figure S3. Electron microscopy image of fibroblast cell from individual F5-II:2. Normal morphology of peroxisome indicated by red arrow.

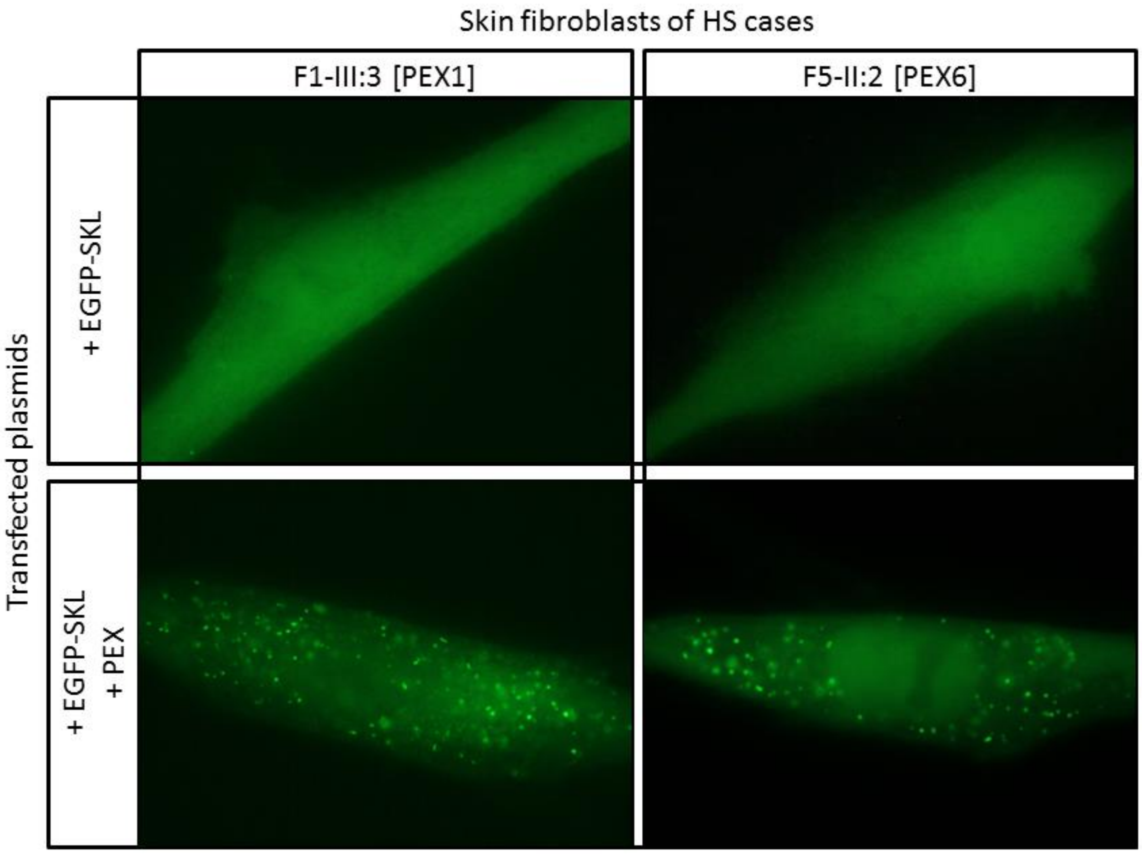

Figure S4. Complementation of peroxisomal defect in HS cells by introduction of PEX1 or PEX6. Skin fibroblasts of HS cases F1-III:3 and F5-II:2 were cultured on 40°C and transfected with a peroxisomal fluorescent marker (EGFP-SKL) and expression vectors containing functional *PEX1* gene copies (for F1-III:3) or *PEX6* gene copies (for F5-II:2). Cells were imaged by fluorescence microscopy and complemented cells (punctate signal) quantified.
